# Comparison of the recovery profile of remimazolam with flumazenil and propofol anesthesia for open thyroidectomy

**DOI:** 10.1186/s12871-023-02104-1

**Published:** 2023-05-02

**Authors:** Ho-Jin Lee, Hyo Bin Lee, Yoon Jung Kim, Hye-Yeon Cho, Won Ho Kim, Jeong-Hwa Seo

**Affiliations:** 1 Department of Anesthesiology and Pain Medicine, Seoul National University Hospital, Seoul, Republic of Korea; 2 Department of Anesthesiology and Pain Medicine, Seoul National University College of Medicine, Daehak-Ro 101, Jongno-Gu, Seoul, Republic of Korea

**Keywords:** General anesthetics, Intravenous anesthesia, Benzodiazepines, Delayed emergence from anesthesia, Flumazenil

## Abstract

**Background:**

Previous studies have consistently reported a slower recovery of consciousness following remimazolam-based total intravenous anesthesia without flumazenil than with propofol. This study aimed to compare the reversal effect of flumazenil on the recovery of consciousness after remimazolam-based total intravenous anesthesia with the propofol recovery profile.

**Methods:**

This prospective, single-blinded, randomized trial included 57 patients undergoing elective open thyroidectomy at a tertiary university hospital. Patients were randomly allocated to receive either remimazolam- or propofol-based total intravenous anesthesia (remimazolam group: 28 patients, propofol group: 29 patients). The primary outcome was the time from the end of general anesthesia to first eye opening (min). The secondary outcomes were the time from the end of the general anesthesia to extubation (min), initial modified Aldrete score measured at the post-anesthesia care unit, length of stay at the post-anesthesia care unit (min), occurrence of postoperative nausea and vomiting during the first 24 h postoperatively, and Korean version of Quality of Recovery-15 score at 24 h postoperatively.

**Results:**

The remimazolam group showed significantly faster first eye opening time (2.3 [interquartile range, IQR: 1.8–3.3] min vs. 5.0 [IQR: 3.5–7.8] min, median difference:—2.7 [95% confidence interval, CI: -3.7 to -1.5] min, *P* < 0.001) and extubation time (3.2 [IQR: 2.4–4.2] min vs. 5.7 [IQR: 4.7–8.3] min, median difference: -2.7 [97.5% CI: -5.0 to -1.6] min, *P* < 0.001). There were no significant differences in other postoperative outcomes.

**Conclusions:**

The planned incorporation of flumazenil with remimazolam-based total intravenous anesthesia provided rapid and reliable recovery of consciousness.

**Supplementary Information:**

The online version contains supplementary material available at 10.1186/s12871-023-02104-1.

## Introduction

Remimazolam is an ultrashort-acting intravenous (IV) benzodiazepine, and in January 2021, South Korea was the second country in the world after Japan to approve its use as a general anesthetic [1]. Owing to its rapid plasma clearance by nonspecific esterases, remimazolam has the advantage of a short context-sensitive half time compared to that of other benzodiazepines [2]. A recent phase III trial in Japan found that the efficacy of remimazolam as a general anesthetic is not inferior to that of propofol, and it has the advantages of not causing injection pain and providing higher hemodynamic safety [3]. However, despite its ultrashort-acting properties, recovery of consciousness after general anesthesia was reported to be slower following remimazolam than following propofol, and remimazolam was associated with a significantly longer time to recovery of consciousness and extubation [3]. Moreover, in the same trial, 9% of patients in the remimazolam group received pre-planned flumazenil owing to delayed recovery (defined as no eye opening observed 30 min after completing remimazolam infusion) [3]. This result was similar to that of a phase III trial conducted in South Korea (Supplemental Table S1, unpublished data from Hana Pharmaceutical, Seoul, South Korea). Another recent study reported that the lapse from the end of remimazolam infusion to extubation was longer than 15 min in 47.7% of patients who received remimazolam-based general anesthesia [4]. This delay in immediate recovery can lead to an increase in medical costs [5].

The use of flumazenil, a benzodiazepine antagonist, is expected to overcome the aforementioned shortcomings associated with remimazolam use [6]. However, there have been no studies on its planned incorporation. In previous studies, flumazenil has been used as a rescue reversal agent in selected patients who had shown delayed recovery [3, 4]. Although the reversal of benzodiazepine effects by flumazenil has been discouraged owing to the risk of re-sedation [7], we expect its routine use to be clinically beneficial considering the shorter duration of the effect of remimazolam compared to that of flumazenil [8, 9]. Moreover, flumazenil has a high safety margin with remarkably few side effects [10]. We planned this prospective randomized controlled trial (RCT) to investigate the reversal effect of flumazenil on the recovery of consciousness after remimazolam-based total intravenous anesthesia (TIVA). We hypothesized that compared to propofol-based TIVA, planned flumazenil use in remimazolam-based TIVA would result in a significant difference in recovery time. Additionally, we aimed to compare perioperative outcomes, including quality of recovery, between two general anesthetics for exploratory purposes.

## Methods

This RCT was approved by the institutional review board (IRB) of our institution (No. H-2105–016-1217) and registered on ClinicalTrials.gov registry before the enrolment of patients (ID: NCT05047939; 17/09/2021). The study was conducted following the Declaration of Helsinki, and all participants provided written informed consent before the study. We designed and reported the study findings following the Consolidated Standards of Reporting Trials recommendations [11].

All adult patients scheduled to undergo elective open thyroidectomy without intraoperative recurrent laryngeal nerve neuromonitoring were screened for their eligibility. Inclusion criteria were adult patients (aged between 18 and 70 years) with an American Society of Anesthesiologists (ASA) physical status of I–II. Patients aged less than 18 years or more than 70 years; those who had an ASA physical status III or higher; those who had a body mass index (BMI) ≥ 40 kg.m^−2^; those who had a history of allergies to medications used in the study protocol; those who received mechanical ventilation for more than 2 h postoperatively; those who received anxiolytics, antipsychotics, rifampicin, succinylcholine, neostigmine, flumazenil, or cyclosporin within 24 h prior to general anesthetics; those who had galactose intolerance, Lapp lactase deficiency, or glucose-galactose malabsorption; those who had underlying systemic diseases or poorly controlled psychiatric disorders that could interfere with the interpretation of the outcome assessments; or those who demonstrated inability to understand the informed consent and study protocol were excluded.

### Randomization and blinding

After providing written informed consent, an anesthesiologist not involved in the study randomly assigned the enrolled patients to the remimazolam or propofol group using blocked randomization in a 1:1 allocation ratio in block size 4 with the R software (version 3.5.1, R Foundation for Statistical Computing, Vienna, Austria). Since anesthesiologist investigators could not be blinded to the group assignment owing to different anesthetic management requirements between the two groups, only patients and outcome assessors in the post-anesthesia care unit (PACU) and ward were blinded to the group assignment.

### Anesthesia and postoperative management

All perioperative management processes besides general anesthesia were similar between the two groups. In the remimazolam group, general anesthesia was induced via continuous infusion of remimazolam (ByFavo, Hana Pharmaceutical, Seoul, South Korea) at 6 mg.kg^−1^.h^−1^ until the patient was unconscious, and it was maintained via a continuous infusion of remimazolam at a rate of 1–2 mg.kg^−1^.h^−1^, keeping the bispectral (BIS) index (BIS Vista, Medtronic, Dublin, Ireland) at 40–60. If the BIS index did not drop below 60, the infusion rate of remimazolam was maintained at 2 mg.kg^−1^.h^−1^. In the propofol group, general anesthesia was induced at the target effect-site concentration of 3.0 ng.ml^−1^ using a target-controlled infusion (TCI) (Injectomat TIVA Agilia; Fresenius Kabi, Germany) with the Marsh pharmacokinetic model of propofol (2% Fresofol, Fresenius Kabi, Korea Ltd, Korea), and it was maintained by adjusting its target effect-site concentration, keeping the BIS index at 40–60. However, to prevent intraoperative awareness, the target effect-site concentration of propofol during the surgery was maintained at a minimum of 2.0 ng.ml^−1^ [12]. In both the groups, a TCI using the Minto pharmacokinetic model of remifentanil was started at the target effect-site concentration of 4.0 ng.ml^−1^ during anesthesia induction to minimize injection pain [13] and titrated to control hemodynamic responses to pain during the surgery. To ensure that patients remained blinded to the group assignment at the induction of anesthesia, the syringe containing general anesthetics and IV route were not exposed to the patients. During anesthesia induction, 0.075 mg of palonosetron and 5 mg of dexamethasone were intravenously administered to all patients. Rocuronium was used as a neuromuscular blocking agent for the induction (0.8 mg.kg^−1^), and in cases of a train of four (TOF) count of 3, 0.15–0.2 mg.kg^−1^ of rocuronium was intravenously administered to maintain a moderate level of neuromuscular block, as monitored by an acceleromyography device (Intellivue NMT module, Philips Healthcare, Amsterdam, Netherlands) during surgery. Intraoperative hypotension defined as a mean blood pressure below 65 mmHg was corrected by administering an IV vasopressor (ephedrine or phenylephrine). With the initiation of strap muscle suturing, 1 g acetaminophen was intravenously administered over 15 min, and efforts were made to maintain the BIS index between 50 and 60 by titrating the infusion rate of propofol or remimazolam. The continuous infusions of general anesthetics were ended when skin closure was completed, after which 2 mg.kg^−1^ of sugammadex was intravenously administered according to the TOF count to reverse neuromuscular blockade. Additionally, in the remimazolam group, 0.2 mg of flumazenil was intravenously administered immediately after administering sugammadex. Extubation was performed if the following conditions were satisfied: eye opening to verbal command, TOF ratio ≥ 1.0, adequate spontaneous ventilation, and hemodynamic stability. If eye opening was not observed after 7 min from the initial administration of flumazenil, then 0.2 mg of additional flumazenil was intravenously administered.

During the PACU stay, 50 μg of IV fentanyl was used as the first-line rescue analgesic and 30 mg of IV ketorolac was used as an alternative if patients complained of postoperative nausea and vomiting (PONV). IV metoclopramide 10 mg was used as a rescue antiemetic in the PACU. PACU nurses, blinded to the group assignment, measured the modified Aldrete score at 5-min intervals and evaluated consciousness to identify re-sedation. Patients with modified Aldrete scores of 9 or higher were discharged to the ward [14].

In the ward, 30 mg of IV ketorolac was used as the first-line rescue analgesic and 4 mg of IV ondansetron was used as a rescue antiemetic. The administration of rescue analgesic and antiemetic in the PACU and ward was determined by attending anesthesiologists or surgeons who were blinded to the group assignment. Oral ibuprofen 200 mg was routinely administered at 8-h intervals beginning on the morning of postoperative day 1.

### Outcome measures

Demographic and intraoperative characteristics were recorded, including age, sex, height, weight, BMI, ASA physical status, Apfel score [15], type of operation, duration of surgery (min), and intraoperative remifentanil consumption (μg). Additionally, the patients completed the Korean version of the Quality of Recovery-15 (QoR-15 K) questionnaire the day before surgery with the help of an investigator blinded to the group assignment [16].

The primary outcome was the time from the end of general anesthesia to first eye opening (min). After the anesthetic infusion ended, the investigator called the patient by name to open their eyes. Prior to surgery, the investigator educated patients to open their eyes when they could hear their name being called. The secondary outcomes were the time from the end of the general anesthesia to extubation (min), initial modified Aldrete score measured within 5 min of arrival at the PACU [14], length of PACU stay (min), occurrence of PONV during the first 24 h postoperatively, and QoR-15 K score at 24 h postoperatively. Intraoperative hemodynamic and anesthetic depth stabilities measured based on median performance error (%), median absolute performance error (%), and wobble (%) were also compared [17]. Data on intraoperative mean blood pressure and BIS index were retrospectively extracted from electronic medical records (EMRs). Blood pressure was automatically recorded every 2.5 min and the BIS index every 1 min in our EMRs. The last preoperative blood pressure measurement by ward nurses was used as a reference value for mean blood pressure, and the BIS reference value was set to 50. We also investigated intraoperative vasopressor use, time from the end of general anesthesia to the end of vital sign monitoring (min) in the operating room, BIS value at the end of the administration of general anesthesia (BIS value at the end of skin closure), administration of rescue analgesics and antiemetics, and length of hospital stay. The outcome assessor (research nurse) who was blinded to the group assignment evaluated the postoperative outcome in the PACU and ward.

### Statistical analysis

In the phase III trial conducted in Japan, the mean (standard deviation [SD]) time from the end of general anesthesia to first eye opening was 10.3 (5.1) min in the propofol group [3]. In a study with midazolam, administration of flumazenil reduced the time from the end of general anesthesia to first eye opening by 60% (from 14.9 min to 6.0 min) [18]. Based on those results, we would require a sample size of 28 in each group to achieve 80% power to detect group differences using the Mann–Whitney U test, with a two-sided alpha of 0.05.

Normality of the distribution of continuous variables was determined using the Shapiro–Wilk test. Continuous data are reported as the mean (SD) or median (interquartile range [IQR]) and were compared between the two groups using the independent t-test or Mann–Whitney U test, respectively. Categorical data are presented as frequencies or percentages and were compared between the two groups using the chi-square test or Fisher’s exact test according to their expected counts. The effect sizes and their 95% CIs were also calculated. The Bonferroni correction was applied for multiple comparisons of variables related to the recovery speed of consciousness other than the primary outcome.

Statistical analyses were performed using R software, version 3.6.1 (R Foundation for Statistical Computing, Vienna, Austria). All statistical tests of hypotheses were two-sided, and *P* < 0.05 was considered statistically significant, except for the repeated measured variables.

## Results

**Fig. 1:**
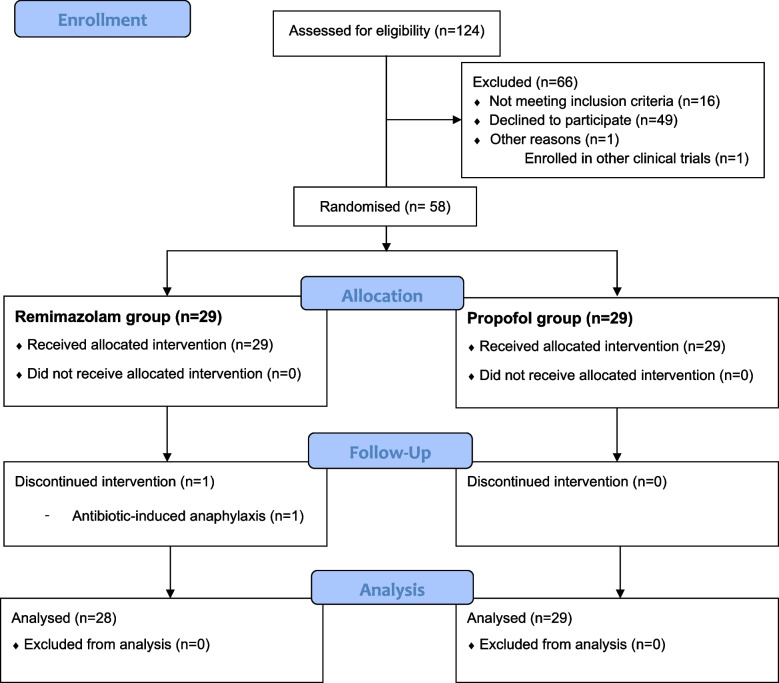
CONSORT diagram of patient recruitment

A total of 124 patients were assessed for eligibility from December 2021 to July 2022 and 58 patients were enrolled and randomly allocated to the remimazolam and propofol groups (Fig. 1). After randomization, one patient in the remimazolam group was excluded due to antibiotic-induced anaphylaxis during the induction of anesthesia and 57 patients were included in the analysis. There was no significant between-group difference in patients’ baseline characteristics (Table 1).

**Table 1:** Baseline characteristics of the remimazolam-based and propofol-based total intravenous anesthesia

Characteristics	Remimazolam group (*n* = 28)	Propofol group (*n* = 29)	*P*-value
Age, years	45 (13.4)	51 (12.1)	0.090
Female sex	21 (75.0)	19 (65.5)	0.622
Height, cm	160.6 (157.2–168.4)	158.4 (153.8–167.0)	0.458
Weight, kg	61.8 (55.2–72.2)	57.5 (52.7–68.0)	0.131
BMI, kg m^−2^	24.3 (22.8–26.0)	22.6 (20.9–25.3)	0.112
ASA physical status, I/II	16 (57.1)/12 (42.9)	17 (58.6)/12 (41.4)	> 0.999
Preoperative QoR-15 K score	146 (140–150)	145 (135–150)	0.280
Apfel score, 0/1/2/3, n (%)	1 (3.6)/5 (17.9)/21 (75.0)/1 (3.6)	1 (3.4)/8 (27.6)/19 (65.5)/1 (3.4)	0.855
Type of surgery			0.358
Total thyroidectomy	9 (32.1)	7 (24.1)	
Right thyroid lobectomy	14 (50.0)	12 (41.4)	
Left thyroid lobectomy	5 (17.9)	10 (34.5)	
Duration of surgery, min	85 (70–98)	85 (75–105)	0.689
Intraoperative remimazolam, mg	175 (144–208)	-	
Intraoperative propofol, mg	-	800 (603–942)	
Intraoperative remifentanil, μg	660 (536–946)	698 (558–889)	0.949

Values are mean (SD), median (IQR) or number (proportion). *IQR* interquartile range, *BMI* body mass index, *ASA* American Society of Anesthesiologist, *QoR-15 K* Korean version of the quality of recovery-15

**Fig. 2:**
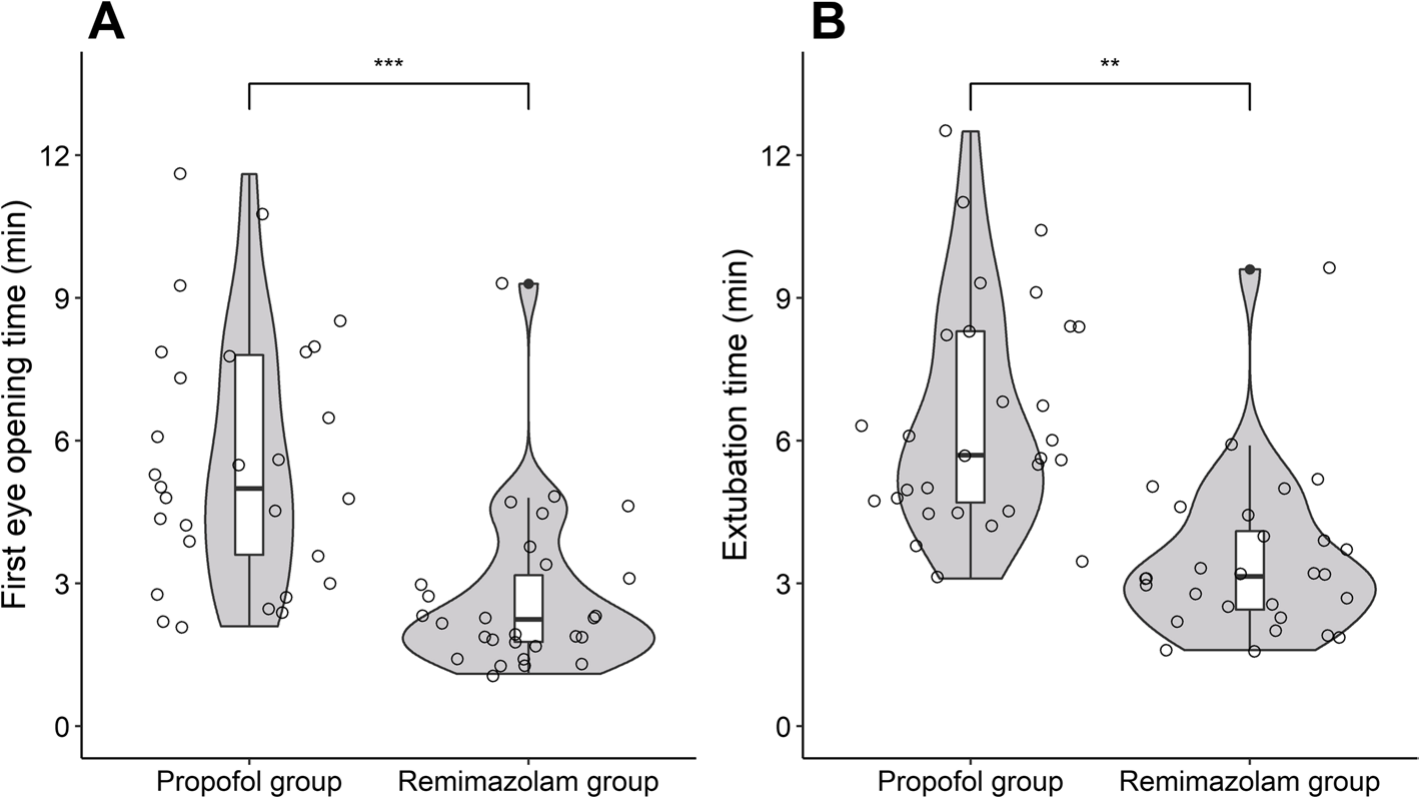
Between group comparisons of the time from the end of the general anesthesia to first eye opening (**A**) and extubation (**B**). The box plot shows the median and interquartile range of time in the remimazolam (*n* = 28) and propofol (*n* = 29) groups. Upper and lower whiskers are maximum and minimum values excluding outliers, respectively. The scatter plot (round symbols) shows the individual data points. The violin plot shows the distribution of the data points for each group. The width of each violin represents the density of the data points. ^**^Adjusted *P* < 0.01, ^***^Adjusted *P* < 0.001

Figure 2 presents a comparison of the time from the end of the general anesthetics to first eye opening and extubation between the two groups. The remimazolam group showed significantly faster first eye opening time (2.3 [IQR: 1.8 to 3.3] min vs. 5.0 [IQR: 3.5 to 7.8] min, median difference:—2.7 min [95% confidence interval, CI: -3.7 to -1.5 min], *P* < 0.001) and extubation time (3.2 [IQR: 2.4 to 4.2] min vs. 5.7 [IQR: 4.7 to 8.3] min, median difference: -2.7 min [97.5% CI: -5.0 to -1.6 min], Bonferroni-adjusted *P* < 0.001). In the remimazolam group, additional flumazenil was administered to only one patient (first eye opening time: 9.3 min, extubation time: 9.6 min). The time from the end of general anesthetics to the end of vital sign monitoring was also significantly shorter in the remimazolam group than in the propofol group (4.8 [IQR: 4.0 to 5.7] min vs. 7.0 [IQR: 5.3 to 9.7] min, median difference: -2.2 min [97.5% CI: -5.0 to -1.6 min], Bonferroni-adjusted *P* = 0.002).

We found no significant group differences when comparing outcomes related to intraoperative hemodynamic and intraoperative anesthetic depth stabilities (Table 2). Similarly, there were no significant group differences in postoperative outcomes (Table 3). No patients were diagnosed with re-sedation in the PACU.

**Table 2:** Comparison of the intraoperative outcomes between the remimazolam-based and propofol-based total intravenous anesthesia

Characteristics	Remimazolam group (*n* = 28)	Propofol group (*n* = 29)	Mean or median or risk difference^a^ (95% Confidence interval)	*P*-value
Intraoperative mean blood pressure				
MDPE, %	7.2 (12.7)	-4.1 (11.5)	11.3 (4.7–18.0)	0.005
MDAPE, %	9.4 (7.6–16.2)	11.7 (8.3–14.9)	-0.3 (-3.1–2.9)	0.894
Wobble, %	5.6 (4.6–7.5)	5.9 (4.9–8.0)	-0.5 (-1.9–0.8)	0.412
Intraoperative vasopressor	7 (25.0)	13 (44.8)	-0.20 (-0.44–0.04)	0.109
Intraoperative BIS value				
MDPE, %	4.3 (9.8)	-12.9 (11.8)	17.3 (11.4–23.1)	< 0.001
MDAPE, %	12.0 (8.3–14.0)	16.0 (12.0–22.0)	-4 (-8.0–-2.0)	0.002
Wobble, %	8.0 (4.5–10.0)	10.0 (8.0–13.0)	-3.0 (-5.0–-2.0)	0.001
BIS value at the end of surgery	57.7 (3.8)	52.3 (5.0)	5.5 (3.0–7.9)	< 0.001

Values are mean (SD), median (IQR) or number (proportion)

^a^Mean or median or risk differences are expressed as the remimazolam group versus the propofol group. *MDPE* median performance error, *MDAPE* median absolute performance error, *BIS* bispectral index

**Table 3:** Comparison of the postoperative outcomes between the remimazolam-based and propofol-based total intravenous anesthesia

Characteristics	Remimazolam group (*n* = 28)	Propofol group (*n* = 29)	Mean or median or risk difference^a^ (95% Confidence interval)	*P*-value
**In the PACU**				
Initial modified Aldrete score	7 (7–8)	7 (7–7)	0 (0–1)	0.063
Rescue analgesics during the PACU stay	5 (17.9)	8 (27.6)	-0.10 (-0.31–0.12)	0.377
PONV during the PACU stay	0	0		
Length of PACU stay, min	30 (30–34.5)	30 (30–39)	0 (-2–2)	0.653
**In the ward**				
Rescue analgesics during the first 24 h Postoperatively	12 (42.9)	11 (37.9)	0.05 (-0.21–0.30)	0.704
PONV during the first 24 h Postoperatively	3 (10.7)	0	0.11 (-0.01–0.22)	0.067
QoR-15 K score at 24 h postoperatively	125 (110–135)	129 (110–139)	-2 (-10–6)	0.571
Length of hospital stay, days	4 (4–5)	4 (4–5)	0 (0–0)	0.377

Values are median (IQR) or number (proportion)

^a^ Median or risk differences are expressed as the remimazolam group versus the propofol group. *PACU* post-anesthesia care unit, *PONV* postoperative nausea and vomiting, *QoR-15 K* Korean version of the quality of recovery-15

## Discussion

In this study, the planned incorporation of flumazenil in remimazolam-based TIVA led to a significantly faster recovery of consciousness with less variation than that with propofol, and it may be used to mitigate delayed recovery reported in previous studies [3, 6, 19]. Moreover, there were no significant differences in the other perioperative outcomes between the two groups.

When investigating the effect of flumazenil on consciousness recovery time following remimazolam use, some considerations should be kept in mind. First, residual neuromuscular blockade can cause discomfort in patients who recovered consciousness quickly by flumazenil and may have affected our primary outcome. Therefore, we tried to maintain a moderate degree of neuromuscular relaxation during surgery and used sugammadex to avoid residual neuromuscular blockade [20]. Second, because there have been no reports of an appropriate dosing regimen of flumazenil with remimazolam-based TIVA, we decided to wait 7 min from its initial administration before re-administration to completely investigate the effect of its initial fixed dose (0.2 mg). As a result, re-administration was required for only one patient and re-sedation was not needed for any patient during PACU stay. However, since the risk of re-sedation after flumazenil administration cannot be overlooked [21], patients should be closely observed, with the possibility of re-sedation during the immediate postoperative period. Lastly, to reproduce a situation closer to real clinical practice, we tried to maintain the BIS index between 50 and 60 by titrating general anesthetics before the end of surgery. Additionally, we educated patients preoperatively to open their eyes on hearing their names called at the end of anesthesia, as in our previous study with the same primary outcome [22]. This may also explain why our propofol group recovered faster than that in a previous study [3].

One study found that the use of flumazenil led to faster recovery following remimazolam-induced sedation compared to that following the use of a placebo [6]. Similar results have been reproduced elsewhere (median time to full alertness: flumazenil, 3.5 min vs. normal saline, 35 min) [19]; however, both studies included only a small number of patients under sedation (6 or 8 patients) [6, 19]. In a recent report, the planned incorporation of flumazenil in remimazolam-based TIVA showed significantly lesser recovery time than that of propofol in patients undergoing endoscopic variceal ligation under general anesthesia [23]. However, the primary outcome of this study was the success rate of the surgical procedure and not the time of recovery of consciousness, and no muscle relaxant was administered except succinylcholine at the induction of anesthesia [23]. Unlike the aforementioned studies [6, 19, 23], our study mainly focused on the reversal effect of flumazenil in patients under general anesthesia with neuromuscular blockade. Therefore, the time to consciousness recovery and extubation was shorter and had lesser variation than when using propofol. Considering its safety [10] and low cost, flumazenil can be useful as a routine reversal agent in remimazolam-based TIVA.

Despite our best efforts, this study had some limitations. First, we could not compare the differences in the recovery speed of remimazolam with or without the administration of flumazenil. The IRB of our institution pointed to previous studies already demonstrating that the administration of flumazenil accelerates recovery speed with remimazolam-based TIVA [6, 19], suggesting that further exploration could lead to unnecessary enrolment of study subjects. Therefore, we had to revise our study design from three to two groups before receiving IRB approval. Second, although we adjusted the anesthetic doses under BIS guidance, we did not manage to maintain the BIS index below 60 in some patients receiving remimazolam. The EEG-derived hypnotic index has not been validated in remimazolam anesthesia [24]; hence, we had to maintain its maximal dose at 2 mg.kg^−1^.h^−1^ in patients who received remimazolam anesthesia and whose BIS value did not fall below 60 or was close to 60. Assuming that the induction dose (6 mg.kg^−1^.h^−1^) was administered for 2 min and the maximum maintenance dose for 83 min in patients with an average weight of 60 kg in the remimazolam group, the total dose was estimated to be 178 mg, which was similar to the median value of the total remimazolam dose of the remimazolam group in our study. However, since this limitation would have been more unfavorable to the recovery speed in the remimazolam group, it is unlikely to have affected our conclusion. Third, propofol was administered using TCI, which has not yet been approved for use with remimazolam anesthesia in South Korea. Therefore, remimazolam was administered using manually controlled infusion at its recommended dose (1–2 mg.kg^−1^.h^−1^). Lastly, although we stated in the Introduction that delayed immediate recovery could lead to increased medical costs, considering the high cost of remimazolam, the use of flumazenil in remimazolam-based anesthesia would not result in cost savings compared to propofol-based anesthesia.

## Conclusions

The planned incorporation of flumazenil rapidly and reliably reversed the effect of remimazolam anesthesia without any complications. Therefore, flumazenil may be routinely used in this setting to promote recovery of consciousness, rendering the use of remimazolam more advantageous than the use of propofol-based TIVA.

## Supplementary Information

**Additional file 1:** **Supplemental table S1.** Results of the secondary outcomes related to recovery of consciousness after general anaesthesia in the phase III clinical trials of remimazolam in South Korea.

## Data Availability

The datasets used and/or analyzed during the current study are available from the corresponding author upon reasonable request and with permission of the Institutional Review Board of the Seoul National University Hospital.

## Abbreviations

ASA: American Society of Anesthesiologists
BIS: Bispectral
BMI: Body mass index
EMRs: Electronic medical records
IV: Intravenous
IRB: Institutional review board
IQR: Interquartile range
QoR-15K: Korean version of Quality of Recovery-15
PACU: Post-anesthesia care unit
PONV: Postoperative nausea and vomiting
RCT: Randomized controlled trial
SD: Standard deviation
TCI: Target-controlled infusion
TIVA: Total intravenous anesthesia
TOF: Train of four
