# Supplementary material for: Comparison of the recovery profile of remimazolam with flumazenil and propofol anesthesia for open thyroidectomy
Source: BMC Anesthesiol. 2023 May 2;23:147. doi: 10.1186/s12871-023-02104-1 (PMC10152604; doi:10.1186/s12871-023-02104-1)
Supplement: Supplementary file 1 — Additional file 1: Supplemental table S1. Results of the secondary outcomes related to recovery of consciousness after general anaesthesia in the phase III clinical trials of remimazolam in South Korea. [file 12871_2023_2104_MOESM1_ESM.docx]

**Supplemental table S1.** Results of the secondary outcomes related to recovery of consciousness after general anaesthesia in the phase III clinical trials of remimazolam in South Korea

|  | Remimazolam group (n=92) | Propofol group (n=90) |
| --- | --- | --- |
| Time to opening eyes from the end of general anaesthesia, min | 16.27 ± 7.13 | 10.02 ± 5.06 |
| Time to endotracheal extubation from the end of general anaesthesia, min | 17.76 ± 6.61 | 11.22 ± 4.93 |
| Time to decision to discharge from operation room from the end of general anaesthesia, min | 23.07 ± 8.34 | 13.83 ± 4.81 |

Values are expressed as mean ± SD. This is an unpublished data (Study title: A Randomised, Multicentre, Parallel group, Active comparator, Single blind, Phase III Clinical Trial to Evaluate the Efficacy and Safety of HNP 2001 in Surgical Patient undergoing General Anaesthesia) and its use has been approved by the Hana Pharmaceutical, Seoul, South Korea.
